# Designing Physical Education Courses Based on Musical Environment: Using Spinning as an Example

**DOI:** 10.3390/ijerph20010208

**Published:** 2022-12-23

**Authors:** Ying Shuai, Xian Liu, Shao-Shen Wang, Yee Cheng Kueh, Garry Kuan

**Affiliations:** 1 School of Sports Management, Shandong Sport University, Jinan 250102, China; 2 Unit of Biostatistics and Research Methodology, School of Medical Sciences, Universiti Sains Malaysia, Kubang Kerian 16150, Kelantan, Malaysia; 3 Exercise and Sports Science Programme, School of Health Sciences, Universiti Sains Malaysia, Kubang Kerian 16150, Kelantan, Malaysia

**Keywords:** physical education teaching environment, curriculum design, exercise, music

## Abstract

The design of physical education (PE) lessons is an ongoing research project that encompasses elements such as teaching ideas, teaching objectives and teaching methods. Music is regarded as the pinnacle form of beauty and combining it with PE not only improves the effectiveness of PE lessons, but also increases the artistry of PE lessons. The purpose of this study is to examine the combination of music and cycling lessons in order to determine the effectiveness of cycling lessons in a musical environment. For study one, 95 undergraduate students were randomly selected to participate in the experiment in two conditions (*M* age = 20.00 years, *SD* = 1.00 years): (1) with music, (2) without music. For study two, 10 students were randomly selected to cycle in three study conditions: (1) synchronous music, (2) asynchronous fast music and (3) asynchronous slow music. Heart rate (HR), rating of perceived exertion (RPE), Exercise-Induced Feeling Inventory (EFI) and sport performance were measured. There were significant differences in HR, RPE, EFI and exercise performance between the two conditions with and without music, and the group with music performed higher than the group without music. In study two, ratings of perceived exertion were significantly lower for the synchronous music condition at 25, 30 min of the steady state portion of the cycling trials. No significant difference between conditions were found in HR, EFI and sport performance. An innovative fundamental model for teaching physical education courses in a musical environment was developed, including five sections: (1) selection of teaching mode, (2) setting of teaching objectives, (3) teaching process and content arrangement, (4) teaching evaluation and (5) precautions. With the addition of a musical environment, sport performance can be enhanced by triggering students’ emotions and cognition.

## 1. Introduction

### 1.1. Physical Education Environment

The physical education environment is divided into physical and mental environments [1] (pp. 314–318). The physical environment is the material basis for the interaction between physical education and learning, and the efficiency of physical and mental activity depends on an appropriate physical environment. Physical activity is influenced by the physical environment and will affect students’ motor cognition, emotional experience and learning behaviour. The psychological environment of physical education is constituted by the interaction of teachers, teaching materials, students and teaching tools, which, in addition to their physical links, also generate psychological activity [2] (pp. 242–243).

The physical education environment is divided into internal elements and external influences. The internal element is the student’s specific laws of physical and mental development. The external factors are the sum of the general school environment, the specific situation of each school, the objectives of physical education, etc. At the same time, five principles for constructing the physical education teaching environment are proposed, namely the principles of coherence, individualization, convenience and optimization, suitability for the place and subjectivity [3]. By studying the soft environment of education (educational cultural characteristics and educational heritage) and educational environment (educational places and facilities), Sun Yan proposes three main characteristics of the physical education teaching environment in China’s general universities: normality, purification and education. Normativity and purity emphasize that the establishment of the physical education environment must conform to the requirements of physical education and ensure the purity and integrity of this organic whole, and education should make it clear that the physical education environment is compatible with the actual education [4].

The sporting environment has a significant impact on the sense of enjoyment of sport among university students. The sporting environment influences the enthusiasm of sportsmen to participate in sporting activities. A suitable sports environment will promote college students’ conscious and purposeful initiative to play sports and influence the behavior of students so that they participate in sports activities happily and actively under the production of a sense of sports pleasure [5]. The survey on the current state of the physical education environment divided the evaluation of the physical education environment into seven directions: natural factors, teaching facilities, ethos and traditions, classroom teaching atmosphere, interpersonal relationships, size and form of combination and management system. The survey found that the lack and obsolescence of teaching facilities was one of the main factors in students’ reduced motivation to attend classes [6]. The relationship between the physical education environment and physical education interest was investigated and analyzed through four factors: physical education facility environment factor, emotional environment factor, interpersonal environment factor and organizational environment factor, which resulted in the strongest relationship between the physical education facility environment and physical education interest in schools [7]. Teaching platforms such as sound system, video matrix system, screen projection system and environmental control system are added to the teaching environment to create an intelligent and technological modern PE teaching design, which ultimately improves the teaching effect [8].

### 1.2. Research and Practice of Instructional Design

The current research and practice of instructional design has gradually developed different trends: (1) Instructional design has begun to focus on interdisciplinary integration. (2) Instructional design focuses on the combination of modern information technology and cutting-edge educational concepts. The emergence of information technology now brings not only innovations in technology but also thinking about teaching concepts and models. (3) Instructional design has begun to focus on the construction of teaching environments under different factors. (4) Instructional design is increasingly focused on the importance of evaluation as well as methods. The design of teaching and learning in PE courses, like other subjects, has to have its own objectives, methods, models, values, etc. From curriculum design to implementation, it should be centered on student development, focus on the creation of a good teaching environment, pay attention to students’ psychological changes and emotional experiences and improve students’ learning autonomy ([9], p. 181).

The researcher proposes to integrate the idea of tea culture with the design and reform of physical education. She explains the essence of tea culture in cultivating the mind and body and discusses that the reform of physical education teaching design should start from the psychological growth and development of students, summarizing the significance of the integration of the two, and practicing the reform of teaching to cultivate the comprehensive development of moral, intellectual, physical and aesthetic talents [10]. The reform of physical education teaching design in universities is proposed from the perspective of ‘performance’, which includes dramatic effect: setting up competition teaching situations to stimulate students’ interest; role effect: setting up different roles to promote students’ development; and stage set: setting up the venue and awakening students’ enthusiasm for performance [11]. Research on the application of IoT technology to a physical education teaching platform had some interesting results. Experimenting in a volleyball course, the comparison found that the platform establishment made this highly theoretical and practical course establish a good interaction pattern between teachers and students [12].

The researcher analyses the developmental advantages of using physical education games in teaching and describes how games can be used rationally for teaching and learning. It is proposed that the content of physical education games should be novel, consider the differences of students and design physical education games that meet the teaching purpose according to the existing venue equipment to improve the effectiveness of physical education [13]. The empirical study of volleyball courses in colleges and universities using the flipped classroom concludes that students’ technical skills and attitudes towards sport have improved under the flipped classroom model compared to the traditional teaching model [14]. Reform and innovation in the physical education curriculum have been increasingly valued in the context of the integration of different cultures and technologies. The design of the curriculum has shifted from conceptual and technical changes to focus on students’ attitudes and emotional changes.

### 1.3. Music and Sport

A common feature of music and sport is the strong sense of rhythm, which controls and induces the frequency, amplitude and psychological state of the movements of the exerciser in the process of exercise. Exercise physiology also mentions that humans develop unique patterns during their life activities, such as the ‘rhythm’ of the organism, which is controlled by the neural response of the brain. The activity of the perceptual and motor systems, as well as the physiological functions of the organs, are all controlled by the nervous system, which determines that the human being is itself a rhythmic being [15] (pp. 172–216). Rhythm, as a universal modality, appears in nature and in social activity with characteristic regularity and cyclical movement changes. Simply put, any behavior that arises is accompanied by a rhythm that takes place; the speed of movement, the magnitude of movement and the strength of muscular exertion are all expressions of regularity. For the athlete, rhythm is the ability to present an orderly variation in the timing and strength of the completed movement during the movement [16].

According to Karageorghis, Kuan and Schiphof-Godart [17], the right music can stimulate up to 20% more exercise. The question of the impact of music rhythm on sport was also raised in 2000, and the relationship between the two has been studied over the last few decades. The German musician Neuendemeiter developed the concept of functional music, also known as ‘practical music’, which he argued should be used primarily for practical purposes and to serve society. Music can now be used in a variety of areas such as psychotherapy, emotional regulation and physical rehabilitation [17].

A study of the effect of different tempo music on cyclists’ state of mind found that fast-paced music had a greater effect on subjects’ subjective exertion than slower-paced music, and that the effect was greater in men than in women [18]. The study found that exercise fatigue was effectively reduced when the rhythm of the music matched the rhythm of the exercise [19]. The 30-min fast-tempo music was the best choice to relieve exercise and mental fatigue [20]. Music and sport share the same characteristic: rhythm. Musical rhythm can help athletes train their sense of rhythm in movement speed, improving the perception of movement and further improving athletic performance [21].

From a physiological point of view, musical rhythms also have a positive effect on the cardiovascular and respiratory systems of athletes. The effect of music on HR (heart rate) and HRV (heart rate variability) was monitored either during moderate-intensity fixed speed running or walking with or without music in a single exercise session. It was found that the supplementation of music stimulated the exercise benefits of positive emotions, while also reducing the induction of negative emotions over time [22]. Both slow and fast music affected the magnitude of heart rate variability during a fixed-speed running exercise condition. With slow music stimulation, the number of high heart rates increased, and the magnitude of the change was noticeable, especially in professional athletes who were extremely sensitive. With fast music, the heart rate of both professional and non-professional athletes tended to be high and steady, especially when the rhythm of the music matched the rhythm of the exercise and the subjects’ heart rate exercise lasted longer [23].

When music was used in an intervention on basketball players’ fluency, it was found that the athletes’ shooting performance all improved [24]. The use of music in track and field training improved the performance of female youths in the long jump by 7 cm with musical accompaniment [25]. In summary of the effects of 15 min, 30 min relaxation music and fast tempo music on the athletic mental fatigue of athletes in five different sports (gymnastics, trampoline, fencing, diving and synchronized swimming), it was found that 30 min of fast-tempo music improved the subjective fatigue of the athletes, while 30 min of relaxation music had a more pronounced improvement in the physiological effects and was the most popular [20]. By completing 10 km of endurance-type exercise to music in the key of C (calming), D (invigorating) and no music, the performance and emotional changes of the athletes were recorded. The study found that the different types of music interventions had significant effects on performance, with music in the key of D being more beneficial in terms of performance, pleasure, positive mood and fluidity, while music in the key of C was more effective in reducing fatigue, anxiety and depression [26]. Boxer Audley Harrison used music to regulate his athletic state, stimulate energy and relieve competition anxiety and other tensions at the 2000 Olympic super-heavyweight boxing tournament, eventually becoming the first British boxer to win an Olympic gold medal in that category [27]. The use of music in international competitions is also well established. In the 2008 Beijing Olympics, swimmer Michael Phelps was challenged by Koudinov after winning eight gold medals for taking off his headphones just two minutes before the competition as a violation of competition rules and published an article suggesting that listening to pre-competition music could lower an athlete’s breathing rate and make a positive impact. The article suggests that listening to music before a race can lower the breathing rate of athletes, increasing their blood oxygen levels and improving their performance [28]. In a study on the effects of running in triathletes, when the music was at the same pace as the exercise, it was found that motivational music lowered blood lactate and reduced oxygen consumption in athletes compared with no music [29]. In addition, the psychological and physiological effects of synchronous music on running performance in hot and humid conditions showed that synchronous music increased the time-to-exhaustion, lowered the ratings of perceived exertion and reduced the heart rate among the healthy male runners [30].

A study of the emotional effects of listening to music on 107 competitors in a competitive event found that 44.86% of the competitors listened to music to reduce their anger and 44.12% listened to music to reduce their pre-competition stress. The results suggest that listening to music helps to increase exercise levels and distracts from fatigue, anxiety and anger. It also helps to stimulate one’s exercise level and improve performance [31]. It was found that string music at 60–80 beats per minute was more effective in combating fatigue and reducing anxiety [32]. When exercisers were asked to listen to waltz music at 60–65 beats per minute during 20 min of cycling at 70% of maximum oxygen uptake, a significant reduction in subjective exertion and fatigue was found. Different music content also affected the subjects, and it was found that listening to soft music for 1 min followed by 2 min of power cycling increased the speed of exercise. A study of athletes’ self-confidence showed that those who listened to music before the race had significantly higher levels of self-confidence than those who did not listen to music [33].

In a study with asynchronous music, Simpson et al. (2006) used BRUMS in a study of 400 m sprint performance to show that synchronized music had a positive effect on non-specialist anaerobic endurance performance [34]. Bacon C et al. measured maximal oxygen uptake by combining a power cycling program with music in an experiment with asynchronous music and found that asynchronous slow music was more effective in reducing oxygen consumption [35]. Berghe et al. [36] found that physical education through musical accompaniment had a significant positive impact on classroom satisfaction. Digelidis et al. [37] showed that the use of background music had a potentially positive impact on students’ classroom satisfaction and intrinsic motivation and that the use of music in the regular curriculum appeared to be a relatively simple and convenient intervention to help teachers increase students’ motivation and engagement enthusiasm for participation.

Beyond controlled experimental or quasi-experimental studies, the use of pedagogically valid designs in research on the impact of music on athletic performance is exceedingly restricted. In the present study, an attempt was made to add to the existing literature by exploring the relevance of music in relation to cycling performance in students. This study’s secondary objective was to investigate the design model of music for teaching and learning in physical education classes. This study conducted a two-part music intervention for student classroom rides. The variables were: with and without music intervention; synchronous music intervention and asynchronous music intervention. We hypothesized that: (1) with music leads to a significant change in heart rate and an increased exercise effect, producing a more positive psychological effect than without music; and (2) synchronized music causes a significant change in heart rate, a lower level of subjective exertion and more energy expenditure than asynchronous fast and asynchronous slow music, producing a more positive psychological effect.

## 2. Materials and Methods

### 2.1. Participants

After getting approval from the institution’s research committee and signed consent, ten classes were selected as experimental participants to be taught the cycling option. A total of 95 undergraduates students volunteered, 75 males and 20 females, age ranged from 20 to 22 years (*M* age = 20.00 years, *SD* = 1.00 years). Due to the use of a repeated-measures approach, it was not anticipated that cycling ability would influence the results.

### 2.2. Experimental design

Prior to the start of the formal experiment, participants made an initial visit to the laboratory to determine their maximum heart rate and to familiarise them with the cycle ergometer and the workout routine. HR determined the intensity level of the exercise. As this intensity corresponds to moderate exercise, physiological responses are expected to attain a steady state at 80% of maximum HR.

For study one, 95 students were randomly assigned into two research conditions: (1) with music and (2) without music. For study two, 10 students were randomly selected to cycle in three research conditions: (1) synchronous music (130 bpm), (2) asynchronous fast music (137 bpm) and (3) asynchronous slow music (123 bpm). Ninety-five participants performed a 30-min all-out cycle in the first two conditions. Ten participants maintained a cadence of 65 rpm for 30 min throughout each bout; a full pedal rotation occurred at 65 rpm in synchronicity with a 130-bpm tempo, with a semi-revolution of the pedals corresponding to each beat of the music. The resistance was fixed for each ride for all the participants.

### 2.3. Auditory Stimuli

The music used in this study is divided into fast-paced music while the type of music used is electronic music. Through consultation with the instructors from the cycling course, electronic music producers and the relevant literature it is clear that cycling is best performed in an up-tempo music environment. This type of music has a tempo of 120–140 bpm (beats per minute), which helps to increase the production of excitatory hormones during exercise.

For the preliminary selection of the music, a total of 150 students aged 20–22 years from the school were provided with three examples of each of the 18 genres of electronic music selected from the Beat Port website that is suitable for exercise at 120–140 bpm. From there, they choose the three categories they like best. For the first, second and third choices, a score of 3, 2 and 1 were given respectively, and the total score was calculated for each genre. The course design music repertoire was selected from the three highest-scoring genres. The three highest-ranking categories were House (sharp drums, simple melody; 120–130 bpm), Psytrance (clear rhythm, rich content 130–155 bpm), Bigroom (heavy bass, regular beat 126–132 bpm) [38].

### 2.4. Dependent Variables

#### 2.4.1. Heart Rate (HR)

A physiological indicator of cardiovascular responsiveness. Heart rate changes are used to monitor the safety of the exercise process, and the results can reflect the level of muscle energy [39]. During exercise, the heart rate was measured from the 5th minute onwards. The heart rate was measured every 5 min for a total of 6 times during the experiment, and the trend of heart rate changes during exercise was observed and recorded.

#### 2.4.2. Rating of Perceived Exertion (RPE)

The RPE scale is a well-established subjective measure of exercise intensity and is the overall internal exertional sensation of the exerciser. In this study, the Borg from 1 to 10 scale, modified to fit the cycling exercise formulation, was used [25]. During exercise, the subject was asked to verbally state the level of subjective exertion from the 5th minute onwards, and a total of 6 measurements were taken during the experiment and the changes in subjective force perception during exercise were analyzed.

#### 2.4.3. Exercise-Induced Feeling Inventory (EFI)

Tests the direct psychological impact of physical exercise. The theoretical premise underlying the application of this questionnaire is that physical activity or exercise can be stimulating for exercisers to produce different emotional states: (1) Energy is restored and stimulated; (2) mind and body are calm; (3) a state of good or bad emotional arousal is produced; (4) fatigue is reached. In this study, at the end of each group of the experiment subjects will fill in the Exercise-Induced Mood Questionnaire to reflect their emotional state after exercise [40].

#### 2.4.4. Cycling Distance

After 30 min of riding at full power, the participants’ exercise distance was recorded and the difference in exercise performance between the two groups with and without music was analysed [41].

#### 2.4.5. Energy Consumption

The amount of energy required to maintain the body’s activity over a period of time. The unit is Kcal. Under the same conditions of time, pedaling frequency and resistance, the differences in the participants’ energy expenditure were examined using a human exercise energy monitor (ActigraphwGT3X-BT) in a test with three types of music: synchronous, asynchronous fast and asynchronous slow music [42].

### 2.5. Equipment

The experimental equipment is divided into spinning bikes, measuring instruments and musical instruments, see Table 1 below.

### 2.6. Experimental Phase

Minimum of 48 h and maximum of 7 days passed between the initial visit and the experimental session. Participants perform a 30-min all-out ride with and without music. Using a digital display screen, participants maintained the prescribed pedal rate under synchronous and asynchronous music circumstances. For the synchronized music and metronome settings, participants were told to match their pedal frequency to the beats of the relevant aural stimuli. In these circumstances, no visual input was offered. The pedal cadence was continuously recorded and monitored to ensure that any significant variations (2 rpm) from the specified cadence were corrected quickly. RPE and HR values were collected continuously throughout the duration of each bout. At 5-min intervals throughout each exercise bout, participants rated their experiences of dyspnea and limb discomfort, in-task affective valence and arousal by indicating a number on the appropriate scale. At the end of the test, participants will fill in an EFI questionnaire to evaluate their mood during the exercise. At the end they record the cycling distance and their energy consumption [38].

To reduce carryover effects, the order of conditions was given at random and completely counterbalanced. Before testing, participants were instructed to desist from any severe physical activity for 48 h. In addition, they were instructed to abstain from caffeine- and alcohol-containing items, as well as foods and beverages (excluding water), for 12 and 3 h, respectively, before each visit.

### 2.7. Data Analysis

Data was processed on a computer using IBM SPSSS statistics 27.0 Mac software. Normal distribution of the numerical data was checked using Kolmogorov–Smirnov and Shapiro–Wilk tests. The normality test results were presented in Table 2 [43]. The majority of the *p*-values were not significant, indicating that the data were normally distributed. Due to the comparison groups being related, paired samples *t*-test and one-way repeated measures ANOVA were used to compare the study conditions in study 1 and study 2 respectively. The significance level was determined to be 0.05, with *p* < 0.05, indicating that the difference was significant, and *p* > 0.05, indicating that the difference was not significant. Comparison of the effects of music, no music and synchronous and asynchronous music on participants’ heart rate, RPE, EFI, exercise distance and energy expenditure.

## 3. Results

### 3.1. Study One: With Music and No Music Conditions

#### 3.1.1. Heart Rate

Conditions have a significant effect on heart rate. Table 3 shows the differences between the heart rate indicators with and without music, using paired samples *t*-tests. From the following results, we can see that there are significant differences in heart rate indicators at 10, 15, 20 and 25 min (*p* < 0.05). The average heart rate of students in the music environment was significantly higher than that in the no music environment in these four time periods, but there was no significant difference in the start and end time indicators.

**Figure 1:**
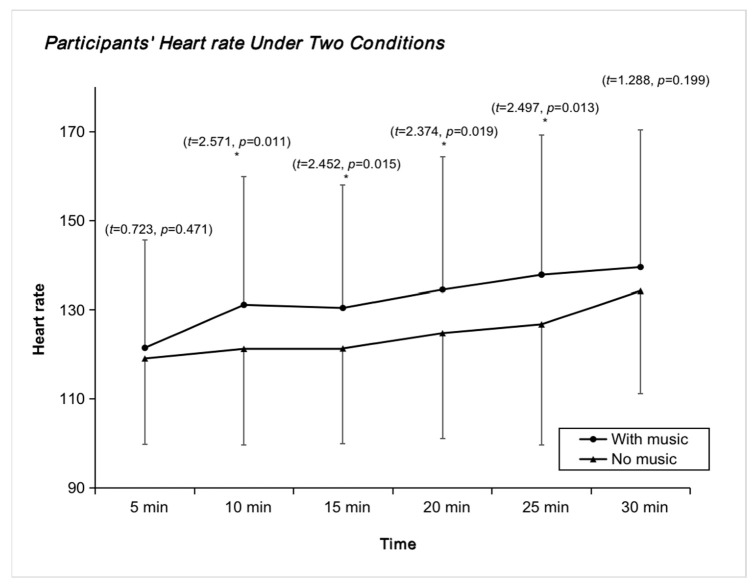
Trend of HR indicators in the presence and absence of music. * *p* < 0.05.

Figure 1 compares the heart rate data between the two environments at different times. It can be seen that with the music intervention, there is a significant increase in heart rate during exercise, indicating that the students maintain good exercise form and further illustrating the need to add music to the intervention during the teaching of spinning.

#### 3.1.2. Rating of Perceived Exertion (RPE)

Conditions have a significant effect RPE. Table 4 shows the differences in the indicators of subjective exertion with and without music. We used paired samples *t*-tests and from the results below, it is clear that there is a significant difference in the indicators of subjective exertion in all time periods (*p* < 0.05) and that the students’ subjective exertion is significantly higher throughout the exercise with music than without music.

**Figure 2:**
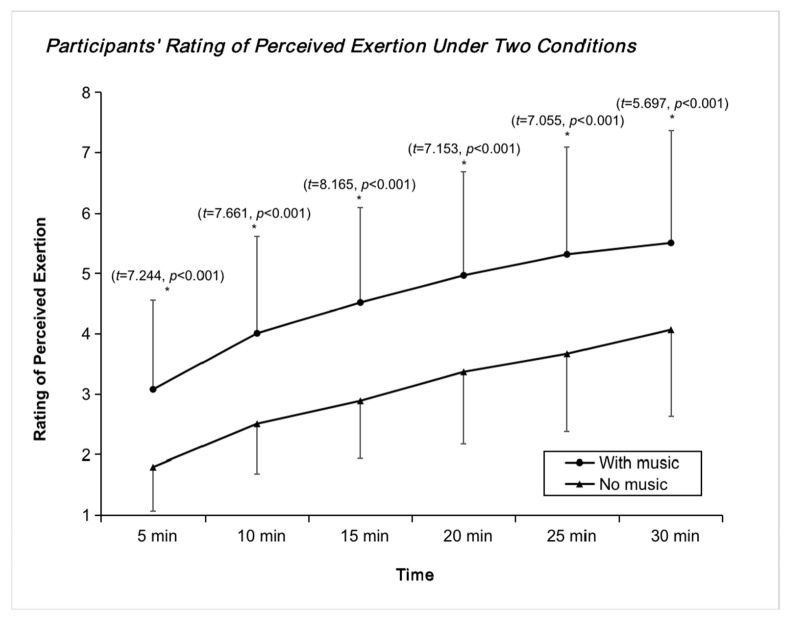
Trend of RPE indicators with and without music. * *p* < 0.05.

Figure 2 compares the RPE data between the two environments at different times. It can be seen that with the music intervention, there was a significant increase in PRE during exercise, indicating a significant increase in self-exercise intensity and an active increase in cycling frequency, further illustrating the need to add music to the intervention during the teaching of spinning.

#### 3.1.3. Exercise-Induced Feeling Inventory (EFI)

Conditions have a significant effect on the EFI. Table 5 presents the analysis of the differences between the post-exercise mood indicators with and without music; using the paired samples *t*-test, the following results show that there are significant differences in revitalization, tranquility, and positive engagement (*p* < 0.05); the students’ vitality and positive engagement were significantly higher in the environment with music than in the environment without music, while the physical and mental calmness was higher without music than with music.

#### 3.1.4. Cycling Distance

Conditions have a significant effect on the cycling distance. Table 6 shows the analysis of the differences between the distance ridden in relation to the indicators with and without music at the end of the exercise; we used paired samples *t*-test, and from the following results it is clear that there is a significant difference (*p* < 0.05) in the exercise distance indicator, with the distance ridden by the students with music being significantly higher than those riding without music.

### 3.2. Study Two: Synchronous Music, Asynchronous Music

Table 7 shows the results of the one-way repeated measures. ANOVA showed that in the synchronous and asynchronous music interventions, only the RPE was significantly different at minute 25 and 30 min (*F* = 5.427; *F* = 5.429, *p* = 0.014; *p* = 0.016). Bonferroni multiple mean comparisons showed that the 25-min RPE of the synchronous music intervention was significantly lower than that of the asynchronous fast music (*p* = 0.045). However, in the synchronous and asynchronous music interventions, HR, FFI and energy consumption were not significant (*p* > 0.05), See Table 8 and Table 9.

## 4. Discussion

The results showed that there were significant differences in the tests and measures of heart rate, cycling distance, RPE and EFI between the two teaching environments with and without music. With the musical intervention, students showed more significant physical recovery, more positive exercise mood, a more active classroom atmosphere and a significant increase in classroom engagement, demonstrating that the use of music in the teaching of dynamic cycling is a viable method for improving classroom quality. It can also be extended for use in other orderly rhythmic physical education courses as a direction for reform and innovation in the physical education curriculum.

The practical results in the same asynchronous music environment show that there is no significant difference in the physiological and psychological effects of the three environments—synchronous music, asynchronous fast music and asynchronous slow music—on students. This suggests that the question of whether the matching of musical rhythm and movement rhythm has an impact on the classroom effect deserves further research.

**Figure 3:**
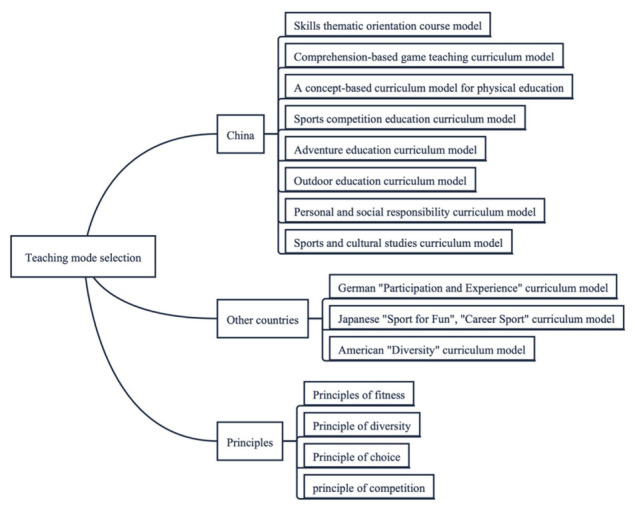
Figure showing the choice of teaching mode for the course.

**Figure 4:**
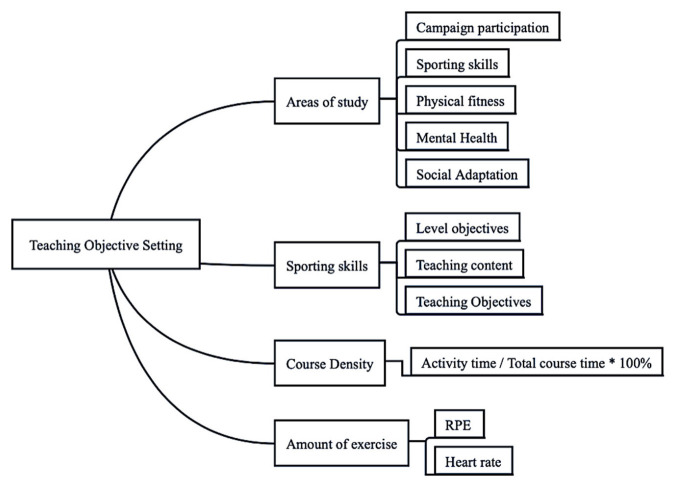
Figure of elements for setting teaching objectives.

**Figure 5:**
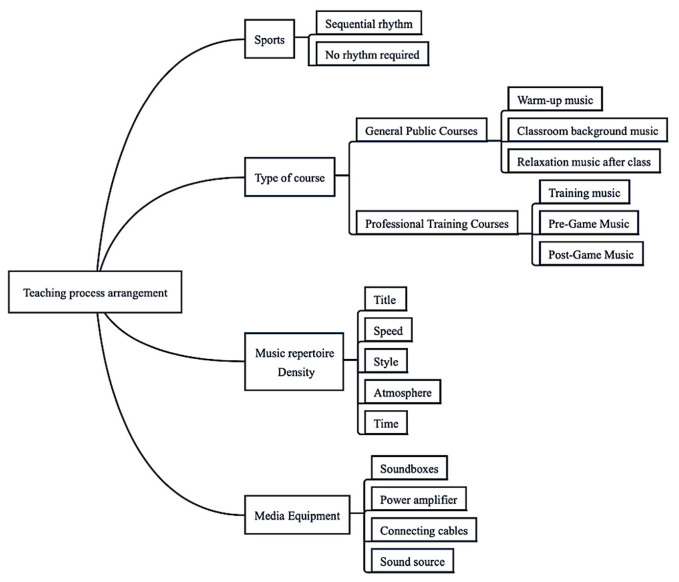
Figure of the PE teaching process under musical conditions.

**Figure 6:**
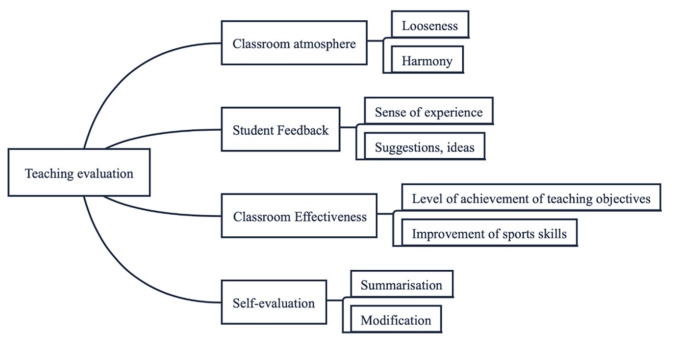
Figure of teaching evaluation.

Based on the results of the study, an innovative fundamental model for teaching physical education courses in a musical environment was developed, including five sections: (1) selection of teaching mode, (2) setting of teaching objectives, (3) teaching process and content arrangement, (4) teaching evaluation and (5) precautions. Figure 3, Figure 4, Figure 5 and Figure 6 show the content in detail respectively.

Current observations show that cycling with music reduces and alleviates the perception of fatigue symptoms compared to conditions without music. Moreover, with music, cyclists can increase their maximum ability in completing the longest distance rides. At the end of the ride, the rider remains in a state of excitement and pleasure and without getting bored with the course, looking forward to the next ride. However, in the synchronous music and asynchronous music test, there were no significant differences in each of the participants’ dependent variables. Future exploration of this section should continue to be explored to create a more complete study.

As seen from the above, the design of a musical environment PE programme will vary according to the actual situation, with music as an important element and other considerations to be considered in the design and implementation process, such as: (1) Consistency with the characteristics and style of the sport; as each sport has its own style and characteristics, the songs chosen should not only be in harmony with the overall movement but also with the movements of each section. (2) Matching the intensity and rhythm of the sport; the intensity of the training and the rhythm of the movements should be clearly defined first. Then appropriate music should be chosen according to the intensity set by the course objectives and the rhythm of the sport. (3) Choosing different rhythms to match; movement exercises can be performed to the same tune throughout the course to develop students’ proficiency or to different tunes, depending on the actual needs. (4) Ensuring the integrity of the repertoire; while the emphasis is on matching the music to the rhythm of the movement, the integrity of the melody of the song should also be ensured, rather than being randomly pieced together, otherwise the content of the music is lost. (5) Cutting the music to ensure that the transition is natural; if only one song is chosen, make sure that the beginning and end are complete; if music of a different rhythm and tune is chosen, cut the music carefully so that the strong and weak beats are in accordance with the rules and there are no mistakes such as empty beats. (6) Consider students’ musicality and musicianship, summarise the class results and continue to complete the teaching design to achieve the best results. Although the design of the musical environment PE programme has proved its efficacy, it is adaptable to diverse circumstances, such as intrinsic and extrinsic factors, personal and situational factors and in different environmental or task-related factors. These variables can affect the program’s outcome (for more information, see Karageorghis et al. [17]). Thus, practitioners are urged to use caution with the variability of the musical environment in order to get the greatest benefits from the PE programme.

## 5. Limitations

There are also some limitations in this study. Firstly, because the experiment could not be changed at will after it was scheduled, the sample size was relatively small, although there were students who wanted to join at a later stage. The lack of sample size made some of the data abnormally distributed. Also, the subjectivity of the measurements was difficult to eliminate, especially in the measurement of RPE, where each participant had different criteria.

It has been demonstrated that each individual has a preferred pace for a given work, and that this differs between individuals. Participants may have been pedalling at a cadence that was not necessarily their preferred pace due to the imposition of a preset pedal frequency. This may have decreased their mechanical efficiency, as they were required to pedal at the set cadence. For instance, professional cyclists prefer a pedal cadence of 80 revolutions per minute [44], which is higher than the 65 revolutions per minute employed in the present study. For future study, it is suggested to recruit more participants from different age groups and changing the speed and music design during cycling.

## 6. Conclusions

The study of the role of music in different areas of sport is not limited to popular sport. It is also worth exploring the role of music in the direction of professional competitions in competitive sports. Professional events such as the 400 m sprint, cycling, weightlifting and ball games are analysed from different perspectives, such as before, during and after the athletes’ races. In addition, physical education teacher practitioners can use the current study’s findings to make more informed designs and choices about how to apply music based on the types of lessons they teach, the target groups and the intended outcomes of the lessons.

When using music, teachers conduct surveys on the preferences of recipients and create song lists belonging to different classes based on the results of the surveys and classroom feedback, while they can be designed by teachers and students together to form special lessons. In class, teachers improve their musical literacy, pay attention to the realization of popular trends and accumulate musical knowledge to provide musical designs for different lessons, while learning to use basic music editing software and playback equipment.

In this study, professional sound equipment is used to ensure the effectiveness of the classroom. In practical application, schools in different areas can select appropriate equipment according to their own conditions and give full play to their greatest advantages. Remote areas can combine local cultural characteristics to select music, and without professional sound can use radio to operate.

In summary, the present findings suggest that a music intervention may enhance performance and trigger emotions and cognitions associated. Further study is required to enable music-related interventions to be accepted by the sports science community on the basis of strong empirical evidence. Specifically, now that the benefits of music have been repeatedly demonstrated in laboratory settings, more ecologically valid and group-based research methods would serve to bolster the knowledge base.

## Figures and Tables

**Table 1:** Table teaching equipment table.

Name	Model	Number of Equipment	Brand
Spinning Bike	LEIKE-P0090	20	Lake
Heart rate monitor	2 pro	20	Xiaomi
Human movement energy monitor	wGT3X-BT	20	Actigraph
DJ Mixer	DDJ-T1	1	Pioneer
Computer	MacBookAir	1	Apple
Music playing software	PRO3.0Mac	1	Traktor
Audio	KP612tweeter	2	JBL
	SRX728S18 bass	2	
Power amplifiers	D350	1	DETON
	D550	2	
Equalisers	231	1	DBX
Mixing consoles	MG12XU	1	Yamaha

**Table 2:** Table Normality test.

	Kolmogorov–Smirnov			Shapiro–Wilk		
	Statistic	*df*	Sig.	Statistic	*df*	Sig.
With music HR 5 min	0.056	76	0.200 *	0.978	76	0.207
With music HR 10 min	0.062	76	0.200 *	0.988	76	0.719
With music HR 15 min	0.068	76	0.200 *	0.973	76	0.102
With music HR 20 min	0.099	76	0.063	0.946	76	0.003
With music Energy consumption	0.073	76	0.200 *	0.960	76	0.017
With music Cycling distance	0.080	76	0.200 *	0.980	76	0.266
No music HR 10 min	0.071	76	0.200 *	0.988	76	0.728
No music HR 15 min	0.089	76	0.200 *	0.983	76	0.420
No music HR 30 min	0.072	76	0.200 *	0.969	76	0.056
No music Energy consumption	0.079	76	0.200 *	0.982	76	0.345
No music Cycling distance	0.089	76	0.200 *	0.976	76	0.164
Synchronous music HR 20 min	0.181	9	0.200 *	0.867	9	0.115
Synchronous music HR 30 min	0.169	9	0.200 *	0.924	9	0.422
Synchronous music Revitalization	0.217	9	0.200 *	0.919	9	0.383
Synchronous music Tranquility	0.159	9	0.200 *	0.943	9	0.609
Synchronous music Physical exhaustion	0.192	9	0.200 *	0.894	9	0.221
Synchronous music Positive engagement	0.143	9	0.200 *	0.98	9	0.964
Asynchronous fast music HR 20 min	0.211	9	0.200 *	0.903	9	0.273
Asynchronous fast music HR 25 min	0.203	9	0.200 *	0.859	9	0.094
Asynchronous fast music HR 30 min	0.185	9	0.200 *	0.936	9	0.543
Asynchronous fast music Energy consumption	0.189	9	0.200 *	0.922	9	0.411
Asynchronous fast music Revitalization	0.197	9	0.200 *	0.914	9	0.342
Asynchronous fast music Tranquility	0.17	9	0.200 *	0.927	9	0.455
Asynchronous fast music Physical exhaustion	0.141	9	0.200 *	0.92	9	0.393
Asynchronous fast music Positive engagement	0.188	9	0.200 *	0.923	9	0.421
Asynchronous slow music HR 10 min	0.216	9	0.200 *	0.83	9	0.044
Asynchronous slow music HR 20 min	0.215	9	0.200 *	0.813	9	0.029
Asynchronous slow music Energy consumption	0.221	9	0.200 *	0.902	9	0.263
Asynchronous slow music Tranquility	0.185	9	0.200 *	0.935	9	0.535
Asynchronous slow music Positive engagement	0.146	9	0.200 *	0.919	9	0.380

* This is a lower bound of the true significance. a Lilliefors Significance Correction.

**Table 3:** Table with and without music for HR differences (Unit: bpm).

	Condition	Mean	SD	*t*-Test	*p*-Value
HR 5 min	With music	121.49	24.075	0.723	0.471
	No music	119.12	19.269		
HR 10 min	With music	131.16	28.749	2.571	0.011 *
	No music	121.27	21.665		
HR 15 min	With music	130.47	27.542	2.452	0.015 *
	No music	121.36	21.344		
HR 20 min	With music	134.47	29.925	2.374	0.019 *
	No music	124.81	23.696		
HR 25 min	With music	137.80	31.383	2.497	0.013 *
	No music	126.77	27.159		
HR 30 min	With music	139.51	30.809	1.288	0.199
	No music	134.21	22.995		

Analyzed by Paired Samples *t*-tests, * *p* < 0.05 significant.

**Table 4:** Table with and without music for RPE differences (Unit: level).

	Condition	Mean	SD	*t*-Test	*p*-Value
RPE 5 min	With music	3.08	1.478	7.244	0.001 *
	No music	1.80	0.724		
RPE 10 min	With music	4.01	1.614	7.661	0.001 *
	No music	2.51	0.829		
RPE 15 min	With music	4.52	1.584	8.165	0.001 *
	No music	2.89	0.957		
RPE 20 min	With music	4.97	1.716	7.153	0.001 *
	No music	3.37	1.190		
RPE 25 min	With music	5.32	1.770	7.055	0.001 *
	No music	3.67	1.283		
RPE 30 min	With music	5.51	1.856	5.697	0.001 *
	No music	4.07	1.438		

Analyzed by Paired Samples *t*-tests, * *p* < 0.05 significant.

**Table 5:** Table with and without music for EFI differences.

	Condition	Mean	SD	*t*-Test	*p*-Value
Revitalization	With music	9.56	2.137	2.824	0.005 *
	No music	8.51	2.805		
Tranquility	With music	7.13	2.776	−2.392	0.018 *
	No music	8.13	2.836		
Physical exhaustion	With music	6.49	3.354	0.807	0.421
	No music	6.11	3.030		
Positive engagement	With music	10.45	2.192	5.408	0.000 *
	No music	8.29	3.134		

Analyzed by paired samples *t*-tests, * *p* < 0.05 significant.

**Table 6:** Table with and without music for riding distance differences (Unit: km).

	Condition	Mean	SD	*t*-Test	*p*-Value
Cycling distance	With music	13.84	1.7718	4.473	0.001 *
	No music	12.78	1.3545		

Analyzed by aired samples *t*-tests, * *p* < 0.05 significant.

**Table 7:** Table synchronous, asynchronous music for variables differences.

Variables	Group	Mean	SD	*F*	*p*-Value
HR 5 min	Synchronous music	77.40	22.950	0.031	0.969
	Asynchronous fast music	79.40	20.791		
	Asynchronous slow music	79.20	19.921		
HR 10 min	Synchronous music	78.60	22.212	0.034	0.967
	Asynchronous fast music	77.30	18.136		
	Asynchronous slow music	79.10	20.339		
HR 15 min	Synchronous music	88.50	27.694	1.284	0.301
	Asynchronous fast music	77.70	18.667		
	Asynchronous slow music	79.60	20.988		
HR 20 min	Synchronous music	95.40	27.846	1.368	0.280
	Asynchronous fast music	94.80	26.038		
	Asynchronous slow music	83.50	18.852		
HR 25 min	Synchronous music	86.60	25.808	0.774	0.419
	Asynchronous fast music	93.60	23.95		
	Asynchronous slow music	82.90	20.808		
HR 30 min	Synchronous music	101.0	26.903	1.595	0.230
	Asynchronous fast music	86.70	17.346		
	Asynchronous slow music	88.50	25.321		
RPE 5 min	Synchronous music	1.22	0.667	1.333	0.291
	Asynchronous fast music	1.56	0.726		
	Asynchronous slow music	1.22	0.441		
RPE 10 min	Synchronous music	1.80	0.919	2.124	0.175
	Asynchronous fast music	2.20	1.033		
	Asynchronous slow music	1.70	0.675		
RPE 15 min	Synchronous music	2.30	1.160	0.945	0.367
	Asynchronous fast music	2.50	1.434		
	Asynchronous slow music	2.0	1.155		
RPE 20 min	Synchronous music	2.80	1.549	2.329	0.126
	Asynchronous fast music	3.10	1.729		
	Asynchronous slow music	2.40	1.174		
RPE 25 min	Synchronous music	2.80	1.033	5.427	0.014 *
	Asynchronous fast music	3.80	1.476		
	Asynchronous slow music	3.10	1.663		
RPE 30 min	Synchronous music	3.0	0.943	5.229	0.016 *
	Asynchronous fast music	4.30	1.703		
	Asynchronous slow music	3.20	1.687		
Revitalization	Synchronous music	9.10	2.183	1.588	0.232
	Asynchronous fast music	10.10	2.514		
	Asynchronous slow music	10.20	3.084		
Tranquility	Synchronous music	7.10	2.283	0.033	0.887
	Asynchronous fast music	7.30	3.713		
	Asynchronous slow music	7.20	2.781		
Physical exhaustion	Synchronous music	6.00	2.828	0.971	0.362
	Asynchronous fast music	6.70	4.138		
	Asynchronous slow music	5.30	2.908		
Positive engagement	Synchronous music	9.70	1.889	0.938	0.410
	Asynchronous fast music	10.60	2.875		
	Asynchronous slow music	10.40	3.026		
Energy consumption	Synchronous music	311.188	115.977	0.528	0.598
	Asynchronous fast music	289.691	93.297		
	Asynchronous slow music	319.865	106.911		

One-way repeated measures ANOVA was used. * p < 0.05 significant.

**Table 8:** Table pairwise comparisons RPE 25 min group differences.

Variables	Group	Mean	SD	*p*-Value	95% CI
Synchronous music	Asynchronous fast music	−1.000 *	0.333	0.045	−1.754, −0.246
	Asynchronous slow music	−0.3	0.26	0.837	−0.889, 0.289
Asynchronous fast music	Asynchronous slow music	0.7	0.335	0.199	−0.058, 1.458

Based on estimated marginal means. CI = Confidence interval. * The mean difference is significant at the 0.05 level. Adjustment for multiple comparisons: Bonferroni.

**Table 9:** Table pairwise comparisons RPE 30 min group differences.

Variables	Group	Mean	SD	*p*-Value	95% CI
Synchronous music	Asynchronous fast music	−1.300	0.496	0.083	−2.754, 0.154
	Asynchronous slow music	−0.200	0.359	1.000	−1.253, 0.853
Asynchronous fast music	Asynchronous slow music	1.100	0.433	0.095	−0.171, 2.371

Based on estimated marginal means. CI = Confidence interval. Adjustment for multiple comparisons: Bonferroni.

## Data Availability

Data is available upon request from the authors.
